# Supplementary material for: Multiple-Vessel-Based Blood Gas Profiles Analysis Revealed the Potential of Blood Oxygen in Mammary Vein as Indicator of Mammary Gland Health Risk of High-Yielding Dairy Cows
Source: Animals (Basel). 2022 Jun 8;12(12):1484. doi: 10.3390/ani12121484 (PMC9219519; doi:10.3390/ani12121484)
Supplement: Supplementary file 1 [file animals-12-01484-s001.zip › animals-1668067-supplementary.pdf]

Table S1. Power Analysis.

Index	Group	Power
1	3	0.187162
2	4	0.268148
3	5	0.346111
4	6	0.419781
5	7	0.488381
6	8	0.551465
7	9	0.608846
8	10	0.660547
9	11	0.706738
10	12	0.747699
11	13	0.78378
12	14	0.81537
13	17	0.887267
14	19	0.920022
15	21	0.943828
16	23	0.960905
17	25	0.973012
18	27	0.981509
19	35	0.996177
20	40	0.998632
21	45	0.999524
22	50	0.999838
23	55	0.999946
24	60	0.999982
25	65	0.999994
26	70	0.999998

Table S2. Basal diet of high-yielding dairy cows.

Item	Amount
Ingredients, %, DM basis	
Corn	13.0
Flaked corn	13.6
Soybean meal	14.4
Molasses	1.31
Beet pulp	6.79
Silage corn	22.8
Alfalfa hay	15.5
Oat hay	4.62
Brewers grain	4.01
Premix[1]	0.55
Premix[2]	3.50
Chemical composition (%, DM)	
Crude protein	15.0
Neutral detergent fiber	37.4
Acid detergent fiber	23.5
Crude ash	7.26
NE_L (Mcal/kg of DM)[3]	1.78

[1]Formulated to provide (per kilogram of DM on average): 150 g of Salt, 310,000 IU of vitamin A, 52,500 IU of vitamin D_3, 2250 IU of vitamin E, 2400 mg of Zn, 16.5 mg of Se, 56.3 mg of I, 600 mg of Fe, 26.3 mg of Co, 1900 mg of Mn and 450 mg of Cu, 50 g of water.

[2]Formulated to provide (per kilogram of DM on average): 0.82% of sodium bicarbonate, 0.41% of calcium hydrogen phosphate, 0.59% of calcium carbonate, 1.23% of calcium salt of palm fatty acid distillate, 0.41% of salt, 0.07% of mold adsorbent, 0.07% of active yeast.

Table S3. Distribution of blood gas profiles in high-yielding dairy cows.

Item[1]	Mammary vein (n=20)						Jugular artery (n=20)						Jugular vein (n=20)					
	Max	Min	Mean	SD	SEM	CV	Max	Min	Mean	SD	SEM	CV	Max	Min	Mean	SD	SEM	CV
Na^+, mmol/L	139	132	137.1	1.76	0.4	1.3	139	133	136.6	1.67	0.38	1.2	138	134	136.2	1.36	0.31	1
K^+, mmol/L	4.2	3.2	3.7	0.22	0.05	5.9	4.3	3.4	3.8	0.21	0.05	5.5	4.6	3.7	4.1	0.23	0.05	5.6
TCO_2, mmol/L	39	30	32.9	1.98	0.45	6	35	28	30.9	1.76	0.4	5.7	34	29	31.5	1.5	0.34	4.8
iCa^{2+}, mmol/L	1.23	1.04	1.14	0.048	0.011	4.2	1.32	1.07	1.19	0.065	0.015	5.5	1.31	1.13	1.22	0.048	0.011	3.9
HCT	0.28	0.21	0.24	0.017	0.004	7.1	0.29	0.22	0.24	0.019	0.004	7.9	0.31	0.21	0.24	0.021	0.005	8.8
HGB, g/dl	9.5	7.1	8.1	0.58	0.13	7.2	9.9	7.5	8.2	0.6	0.14	7.3	10.5	7.1	8.2	0.73	0.17	8.9
pH	7.64	7.46	7.5	0.044	0.01	0.6	7.56	7.38	7.5	0.05	0.011	0.7	7.67	7.38	7.44	0.059	0.014	0.8
pCO_2, mmHg	45.6	37.7	40.9	2.12	0.49	5.2	43.9	32.4	36.8	2.93	0.67	8	49.6	39.6	45	2.22	0.51	4.9
pO_2, mmHg	49	29	36.9	5.46	1.25	14.8	125	72	104.2	11.74	2.69	11.3	39	22	31.2	4.57	1.05	14.6
HCO_3, mmol/L	37.5	29	31.6	1.92	0.44	6.1	33.9	27.2	29.7	1.68	0.39	5.7	33.1	27.9	30.1	1.47	0.34	4.9
BEecf, mmol/L	11	6	8.3	1.56	0.36	18.8	9	5	6.9	1.31	0.3	19	8	4	5.7	1.31	0.3	23
Saturation of O_2 %	87	61	73.6	7.06	1.62	9.6	99	96	98.5	0.83	0.19	0.8	74	39	60.2	9.92	2.28	16.5

[1] TCO_2 = total carbon dioxide concentration; iCa^{2+} = ionized calcium, HCT = hematocrit, HGB = hemoglobin, pH, pO_2 = oxygen pressure, pCO_2 = pressure of carbon dioxide, BEecf = base excess in the extracellular fluid

Table S4. Correlation analysis between blood gas profile of jugular artery (-JA) and variables related with oxidative stress and permeability in milk (-M).

**Items** [1]	**SOD-M**	**pO_2-JA**	**K^+-M**	**Hct-JA**	**HGB-JA**	**oxygen content-JA**	**T-AOC-M**	**Na^+/K^+-M**	**SO_2%-JA**	**K^+-JA**	**Plasmin-M**	**MDA-M**	**LDH-M**
SOD-M	1												
pO_2-JA	0.27 (-0.19-0.64)	1											
K^+-M	0.13 (-0.33-0.54)	0.02 (-0.43-0.45)	1										
Hct-JA	0.03 (-0.42-0.46)	0.03 (-0.42-0.46)	0.2 (-0.26-0.59)	1									
HGB-JA	0.01 (-0.43-0.45)	-0.07 (-0.50-0.38)	0.57 (0.17-0.81)	0.72 (0.41-0.88)	1								
oxygen content-JA	0.01 (-0.43-0.45)	-0.02 (-0.45-0.43)	0.53 (0.11-0.79)	0.74 (0.45-0.89)	0.99 (0.98-1.00)	1							
T-AOC-M	-0.35 (-0.68-0.11)	-0.36 (-0.69-0.10)	-0.07 (-0.50-0.38)	-0.24 (-0.61-0.23)	-0.02 (-0.46-0.43)	-0.01 (-0.35-0.53)	1						
Na^+/K^+-M	-0.07	-0.25	-0.66	0.01	-0.35	-0.31	0.26	1					

	(-0.49-0.39)	(-0.62-0.22)	(-0.85--0.31)	(-0.43-0.45)	(-0.69-0.11)	(-0.66-0.16)	(-0.21-0.63)						
SO_2%-JA	-0.11 (-0.52-0.35)	0.07 (-0.39-0.49)	-0.2 (-0.59-0.27)	0.4 (-0.05-0.72)	0.27 (-0.20-0.63)	0.37 (-0.09-0.70)	0.26 (-0.21-0.63)	0.39 (-0.07-0.71)	1				
K^+-JA	-0.36 (-0.69-0.10)	-0.12 (-0.54-0.34)	-0.36 (-0.69-0.10)	0.06 (-0.39-0.49)	-0.16 (-0.56-0.30)	-0.16 (-0.56-0.30)	-0.07 (-0.49-0.39)	0.27 (-0.20-0.64)	0.02 (-0.43-0.46)	1			
Plasmin-M	-0.78 (-0.91--0.50)	-0.16 (-0.56-0.31)	0.1 (-0.36-0.52)	-0.17 (-0.57-0.29)	-0.03 (-0.46-0.42)	-0.05 (-0.48-0.40)	0.18 (-0.28-0.58)	-0.15 (-0.55-0.32)	-0.18 (-0.58-0.29)	0.08 (-0.38-0.50)	1		
MDA-M	-0.92 (-0.97--0.80)	-0.11 (-0.52-0.35)	0.04 (-0.41-0.48)	-0.1 (-0.52-0.35)	-0.03 (-0.46-0.42)	-0.03 (-0.47-0.42)	0.19 (-0.28-0.58)	-0.1 (-0.52-0.36)	0.01 (-0.43-0.45)	0.32 (-0.15-0.67)	0.84 (0.64-0.94)	1	
LDH-M	-0.9 (-0.96--0.76)	-0.02 (-0.45-0.43)	0.01 (-0.43-0.45)	0 (-0.44-0.45)	0.11 (-0.35-0.53)	0.11 (-0.35-0.53)	0.2 (-0.27-0.59)	-0.02 (-0.46-0.42)	0.07 (-0.38-0.50)	0.29 (-0.18-0.65)	0.83 (0.62-0.93)	0.92 (0.80-0.97)	1

[1] SOD: Super oxide dismutase; pO_2: oxygen pressure; Hct: red blood cell specific volume; HGB: hemoglobin; T-AOC: total antioxidant capacity; SO_2: oxygen saturation; MDA: malonaldehyde; LDH: lactic dehydrogenase.

The bracket is 95% confidence intervals.

Table S5. Correlation analysis between blood gas profile of jugular vein (-JV) and variables related with oxidative stress and permeability in milk (-M).

Items [1]	K^+-M	Hct-JV	HGB-JV	SOD-M	oxygen content-JV	Na^+/K^+-M	K^+-JV	pO_2-JV	SO_2%-JV	T-AOC-M	Plasmin-M	MDA-M	LDH-M
K^+-M	1												
Hct-JV	0.4 (-0.06-0.71)	1											
HGB-JV	0.39 (-0.06-0.71)	0.99 (0.98-1.00)	1										
SOD-M	0.13 (-0.33-0.54)	0.1 (-0.35-0.52)	0.09 (-0.37-0.51)	1									
oxygen content-JV	0.17 (-0.30-0.57)	0.32 (-0.14-0.67)	0.37 (-0.09-0.70)	0.29 (-0.18-0.65)	1								
Na^+/K^+-M	-0.66 (-0.85--0.31)	-0.4 (-0.71-0.05)	-0.41 (-0.72-0.05)	-0.07 (-0.49-0.39)	-0.02 (-0.46-0.43)	1							
K^+-JV	-0.47 (-0.76--0.04)	-0.23 (-0.61-0.23)	-0.22 (-0.60-0.25)	-0.39 (-0.71-0.06)	-0.27 (-0.63-0.20)	0.37 (-0.09-0.70)	1						
pO_2-JV	-0.02	-0.14	-0.16	0.19	-0.03	0.02	-0.09	1					

	(-0.46-0.42)	(-0.55-0.32)	(-0.56-0.30)	(-0.27-0.59)	(-0.47-0.42)	(-0.43-0.46)	(-0.51-0.37)						
SO_2%-JV	-0.02 (-0.46-0.43)	-0.19 (-0.58-0.28)	-0.21 (-0.60-0.25)	0.14 (-0.32-0.55)	-0.06 (-0.49-0.39)	0 (-0.44-0.44)	-0.11 (-0.53-0.35)	0.98 (0.95-0.99)	1				
T-AOC-M	-0.07 (-0.50-0.38)	-0.16 (-0.56-0.30)	-0.17 (-0.57-0.30)	-0.35 (-0.68-0.11)	0.11 (-0.35-0.53)	0.26 (-0.21-0.63)	0.06 (-0.39-0.49)	0.18 (-0.29-0.57)	0.18 (-0.29-0.57)	1			
Plasmin-M	0.1 (-0.36-0.52)	-0.07 (-0.49-0.39)	-0.07 (-0.50-0.38)	-0.78 (-0.91--0.51)	-0.31 (-0.66-0.15)	-0.15 (-0.55-0.32)	0.4 (-0.05-0.72)	-0.06 (-0.49-0.40)	-0.04 (-0.48-0.41)	0.18 (-0.28-0.58)	1		
MDA-M	0.04 (-0.41-0.48)	-0.1 (-0.52-0.36)	-0.09 (-0.51-0.37)	-0.92 (-0.97--0.80)	-0.32 (-0.67-0.15)	-0.1 (-0.52-0.36)	0.34 (-0.12-0.68)	0.01 (-0.44-0.45)	0.05 (-0.40-0.49)	0.19 (-0.28-0.58)	0.84 (0.64-0.94)	1	
LDH-M	0.01 (-0.43-0.45)	0.02 (-0.43-0.46)	0.01 (-0.43-0.45)	-0.9 (-0.96--0.76)	-0.35 (-0.69-0.11)	-0.02 (-0.46-0.42)	0.38 (-0.08-0.70)	-0.06 (-0.49-0.39)	-0.02 (-0.46-0.43)	0.2 (-0.27-0.59)	0.83 (0.62-0.93)	0.92 (0.80-0.97)	1

[1] SOD: Super oxide dismutase; pO_2: oxygen pressure; Hct: red blood cell specific volume; HGB: hemoglobin; T-AOC: total antioxidant capacity; SO_2: oxygen saturation; MDA: malonaldehyde; LDH: lactic dehydrogenase.

The bracket is 95% confidence intervals.

Table S6. Correlation analysis between blood gas profile of mammary vein (-MV) and variables related with oxidative stress and permeability in milk (-M).

**Items[1]**	**pO_2-MV**	**SO_2%-MV**	**SOD-M**	**oxygen content-MV**	**K^+-M**	**Hct-MV**	**HGB-MV**	**Na^+/K^+-M**	**K^+-MV**	**T-AOC-M**	**Plasmin-M**	**MDA-M**	**LDH-M**
pO_2-MV	1												
SO_2%-MV	0.96 (0.89-0.98)	1											
SOD-M	0.83 (0.61-0.93)	0.83 (0.61-0.93)	1										
oxygen content-MV	0.78 (0.52-0.91)	0.78 (0.52-0.91)	0.9 (0.75-0.96)	1									
K^+-M	0.03 (-0.42-0.47)	0 (-0.44-0.44)	0.13 (-0.33-0.54)	0.3 (-0.16-0.66)	1								
Hct-MV	0 (-0.44-0.45)	-0.07 (-0.50-0.39)	0.35 (-0.11-0.68)	0.56 (0.15-0.80)	0.5 (0.08-0.77)	1							
HGB-MV	-0.01 (-0.45-0.43)	-0.07 (-0.50-0.38)	0.34 (-0.12-0.68)	0.55 (0.15-0.80)	0.47 (0.04-0.76)	1	1						
Na^+/K^+-M	-0.13	-0.09	-0.07	-0.25	-0.66	-0.32	-0.27	1					

	(-0.54-0.33)	(-0.51-0.37)	(-0.49-0.38)	(-0.63-0.21)	(-0.85--0.31)	(-0.67-0.15)	(-0.63-0.20)						
K$^+$-MV	-0.47 (-0.76--0.04)	-0.52 (-0.78--0.10)	-0.42 (-0.73-0.03)	-0.55 (-0.80--0.14)	-0.53 (-0.79--0.11)	-0.15 (-0.55-0.32)	-0.13 (-0.54-0.33)	0.32 (-0.15-0.66)	1				
T-AOC-M	-0.42 (-0.73-0.03)	-0.35 (-0.68-0.11)	-0.35 (-0.68-0.11)	-0.37 (-0.70-0.08)	-0.07 (-0.50-0.38)	-0.15 (-0.56-0.31)	-0.13 (-0.54-0.33)	0.26 (-0.21-0.63)	-0.01 (-0.45-0.44)	1			
Plasmin-M	-0.62 (-0.83--0.24)	-0.61 (-0.83--0.23)	-0.78 (-0.91--0.51)	-0.7 (-0.87--0.37)	0.1 (-0.36-0.52)	-0.32 (-0.67-0.14)	-0.32 (-0.67-0.15)	-0.15 (-0.55-0.32)	0.11 (-0.35-0.53)	0.18 (-0.28-0.58)	1		
MDA-M	-0.74 (-0.89--0.44)	-0.78 (-0.91--0.51)	-0.92 (-0.97--0.80)	-0.85 (-0.94--0.65)	0.04 (-0.41-0.48)	-0.33 (-0.67-0.14)	-0.33 (-0.67-0.14)	-0.1 (-0.52-0.36)	0.37 (-0.09-0.70)	0.19 (-0.28-0.58)	0.84 (0.64-0.94)	1	
LDH-M	-0.78 (-0.91--0.52)	-0.78 (-0.91--0.51)	-0.9 (-0.96--0.76)	-0.78 (-0.91--0.51)	0.01 (-0.43-0.45)	-0.21 (-0.60-0.25)	-0.2 (-0.59-0.27)	-0.02 (-0.46-0.42)	0.35 (-0.11-0.69)	0.2 (-0.27-0.59)	0.83 (0.62-0.93)	0.92 (0.80-0.97)	1

[1] SOD: Super oxide dismutase; pO_2: oxygen pressure; Hct: red blood cell specific volume; HGB: hemoglobin; T-AOC: total antioxidant capacity; SO_2: oxygen saturation; MDA: malonaldehyde; LDH: lactic dehydrogenase.

The bracket is 95% confidence intervals.
